# Characterization of Different Types of Epiretinal Proliferations by Synchrotron Radiation-Based Fourier Transform Infrared Micro-Spectroscopy

**DOI:** 10.3390/ijms24054834

**Published:** 2023-03-02

**Authors:** Sofija Andjelic, Martin Kreuzer, Marko Hawlina, Xhevat Lumi

**Affiliations:** 1 Eye Hospital, University Medical Centre, 1000 Ljubljana, Slovenia; 2 CELLS-ALBA, Synchrotron Light Source, 08290 Cerdanyola del Valles, Barcelona, Spain

**Keywords:** retina, epiretinal membrane, proliferative vitreoretinopathy, proliferative diabetic retinopathy, extracellular matrix, macromolecule analysis, oxidative stress, synchrotron radiation-based Fourier transform infrared micro-spectroscopy

## Abstract

Pathological tissue on the surface of the retina that can be of different etiology and pathogenesis can cause changes in the retina that have a direct consequence on vision. Tissues of different etiology and pathogenesis have different morphological structures and also different macromolecule compositions usually characteristic of specific diseases. In this study, we evaluated and compared biochemical differences among samples of three different types of epiretinal proliferations: idiopathic epiretinal membrane (ERMi), membranes in proliferative vitreoretinopathy (PVRm), and proliferative diabetic retinopathy (PDRm). The membranes were analyzed by using synchrotron radiation-based Fourier transform infrared micro-spectroscopy (SR-FTIR). We used the SR-FTIR micro-spectroscopy setup, where measurements were set to achieve a high resolution that was capable of showing clear biochemical spectra in biological tissue. We were able to identify differences between PVRm, PDRm, and ERMi in protein and lipid structure; collagen content and collagen maturity; differences in proteoglycan presence; protein phosphorylation; and DNA expression. Collagen showed the strongest expression in PDRm, lower expression in ERMi, and very low expression in PVRm. We also demonstrated the presence of silicone oil (SO) or polydimethylsiloxane in the structure of PVRm after SO endotamponade. This finding suggests that SO, in addition to its many benefits as an important tool in vitreoretinal surgery, could be involved in PVRm formation.

## 1. Introduction

The growth of pathological tissue on the surface of the retina can cause changes that have a direct consequence on vision. The etiology and pathogenesis of such changes are diverse. Risk factors and pathophysiological processes involved in the formation of nonvascular idiopathic epiretinal membranes (ERMi), membranes in proliferative vitreoretinopathy (PVRm), and neovascular membranes in proliferative diabetic retinopathy (PDRm) are different in many respects [1].

Vitreoretinal interface disorders are conditions where epiretinal proliferation often occurs. A common occurrence in vitreoretinal interface disorders is an epiretinal membrane (ERM), which is a fibrotic membrane over the retina that contracts, wrinkling the underlying retina. Due to the predilection for central localization on the surface of the macula, in the more severe forms of ERM, the vision is often affected because of the disruption of macular anatomy [2]. ERMs are made by non-angiogenic fibroglial tissue [2]. They are composed of bundles of non-cellular extracellular matrix (ECM) proteins as the outermost layer, which is laid upon the inner limiting membrane, and an inner cellular sheet, which can be either single or multi-layered [3]. The membranes comprise different types of cells like retinal pigment epithelial (RPE) cells, glial cells, hyalocites, fibrocytes, fibrous astrocytes and myofibroblast-like cells [4,5,6,7,8]. ECM made from collagen plays a crucial role in ERM construction as well as in cell proliferation and migration [2].

Another nonvascular formation representing a pathological condition of the retina is PVR [9], which represents a process of the growth of membranes on the inner and outer retinal surface—which gradually turn into scars in patients with rhegmatogenous retinal detachment (RRD). It is the major complication following retinal detachment surgery and is also a leading cause of failure in the management of RRD [10]. Histologically, these membranes are fibrocellular sheets composed of ECM and different types of cells, like RPE cells, glial cells, fibroblasts, myofibroblast-like cells, and ECM [9].

PDR is the advanced stage of the ocular manifestations of diabetes mellitus [11]. The disease is hallmarked by neovascularization as a response to ischemia, which at its latest stage changes to fibrovascular and fibrous proliferations on the surface of the retina, leading to tractional retinal detachment and loss of vision [12]. In contrast to the cellular structure of PDRms that has been widely and evaluated in more detail, the nature of the ECM of these membranes is less investigated. The pathological changes occurring in the retina during diabetes mellitus are not fully understood yet.

Despite the fact that the structure of angiogenic and non-angiogenic proliferations on the retina has been precisely analyzed by immunochemical and histochemical methods, so far, there have been very few reports on the analysis of their molecular content and their conformational changes [13]. In this study, we evaluated and compared biochemical differences: protein conformational changes and changes in lipids and carbohydrates among three samples of different types of epiretinal proliferation: ERMi, PVRm and PDRm. To assess bio-macromolecules and to provide their molecular fingerprint for better understanding—among the others—of the ECM proteins in nonvascular and neovascular ocular pathological membranes, we used synchrotron radiation-based Fourier transform infrared (SR-FTIR) micro-spectroscopy. SR-FTIR micro-spectroscopy is a vibrational spectroscopic imaging technique, which has the potential for macromolecule analysis in a single cell, and detects different spectra of proteins [14], lipids [15], carbohydrates, and nucleic acids [16]. Spectral data analysis provides qualitative and quantitative information on the basis of peak shifts, bandwidths, and band intensities.

## 2. Results

The spectra of three different types of epiretinal membranes were analyzed for protein, lipid, nucleic acids, and carbohydrate regions. Additionally, we used principal component analysis (PCA) of the FTIR spectra and concentrated on the first two principal components, PC1 and PC2, at each spectral region.

### 2.1. Lipid Region

**Figure 1:**
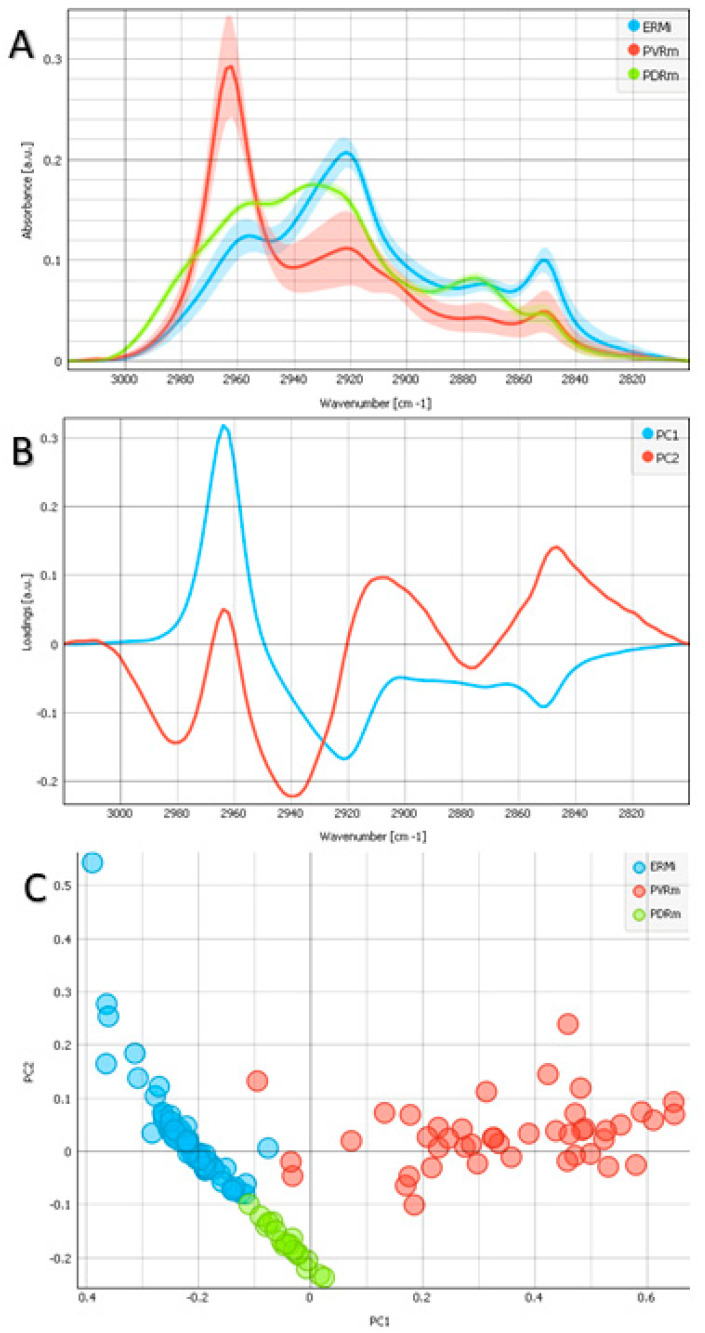
Analysis of the lipid region (2800–3100 cm^−1^). (**A**) Fourier transform infrared micro-spectroscopy (FTIR) average spectra with a standard deviation of examples of 3 different types of epiretinal proliferations: idiopathic epiretinal membrane (ERMi) in blue, membranes in proliferative vitreoretinopathy (PVRm) in red, and proliferative diabetic retinopathy (PDRm) in green. (**B**) Principal component analysis (PCA) loadings of the first two components (PC1 in blue and PC2 in red). (**C**) PCA scores plot denotes the variability associated with the first two components.

Figure 1A demonstrates FTIR average spectra with standard deviations acquired from the lipid region (2800–3100 cm^−1^) [17,18]. A PCA of this region is demonstrated in Figure 1, together with the loadings plots (Figure 1B) and the PCA score (Figure 1C). The analysis shows that the PVRm is pronouncedly different from the others, with PC1 showing the strongest difference and biggest expression of the band with a maximum at 2962 cm^−1^, corresponding to the asymmetric CH_3_ vibration. On the other hand, in PVRm, there is a lower contribution at 2920 cm^−1^, corresponding to the asymmetric vibration of CH_2_, and 2850 cm^−1^, corresponding to the symmetric vibration of CH_2_, both being more expressed in ERMi and PDRm. From the spectra, it can be seen that ERMi showed the highest absorbance at 2922 cm^−1^, corresponding to the presence of asymmetric vibration of CH_2_ and also at 2851 cm^−1^, corresponding to symmetric vibration of CH_2_. In comparison with ERMi, PVRm had fewer contributions of the same wavenumbers, 2922 cm^−1^ and 2851 cm^−1^, and corresponding constituents, as visible from the difference in absorbance intensity. PDRm showed maxima at 2956 cm^−1^ and 2933 cm^−1^ and smaller ones at 2876 cm^−1^ and 2851 cm^−1^, corresponding to the asymmetric vibration of CH_3_ and CH_2_ and the symmetric vibration of CH_3_ and CH_2_, respectively.

### 2.2. Protein Region

**Figure 2:**
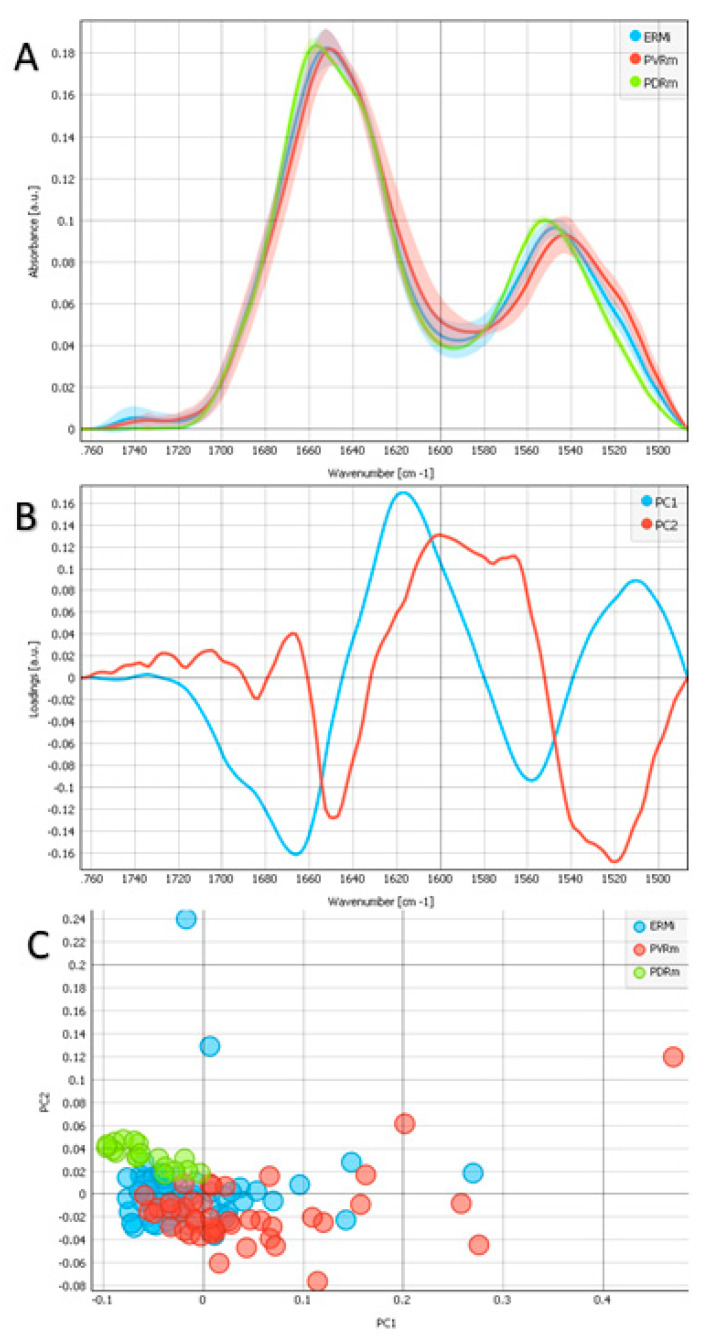
Analysis of the spectral regions of Amide I, Amide II and carbonyl region, including the ester groups (1485–1765 cm^−1^) for examples of 3 different types of epiretinal proliferations (ERMi-blue, PVRm-red, PDRm-green). (**A**) Average FTIR spectra, including standard deviation. (**B**) PCA loadings of the first two PCA components (PC1 in blue and PC2 in red). (**C**) PCA scores plot denotes the variability in the first two components.

The Amide I and Amide II regions, including the ester group (1485–1765 cm^−1^), are demonstrated in Figure 2 [17,18,19]. We noted the Amide I band maxima at 1650 cm^−1^ for PVRm, 1653 cm^−1^ for ERMi, and 1657 cm^−1^ for PDRm. We also noted differences in the Amide II band positions with maxima at 1543 cm^−1^ for PVRm, at 1547 cm^−1^ for ERMi, and at 1553 cm^−1^ for PDRm. The first component of the PCA (Figure 2B) indicated the contributions of 1666 cm^−1^ and 1558 cm^−1^, being the most expressed in PDRm, with the first associated with the turns and loops secondary structure in proteins and the second with the NH bend and C-N stretch of Amide II in proteins. The second component of the PCA (Figure 2B) showed contributions of the bands at 1520 cm^−1^ and 1650 cm^−1^, with the first being associated with tyrosine proteins and the second with the α-helix structure, being more expressed in PVRm and ERMi than in PDRm.

### 2.3. Protein Region Band Deconvolution

**Figure 3:**
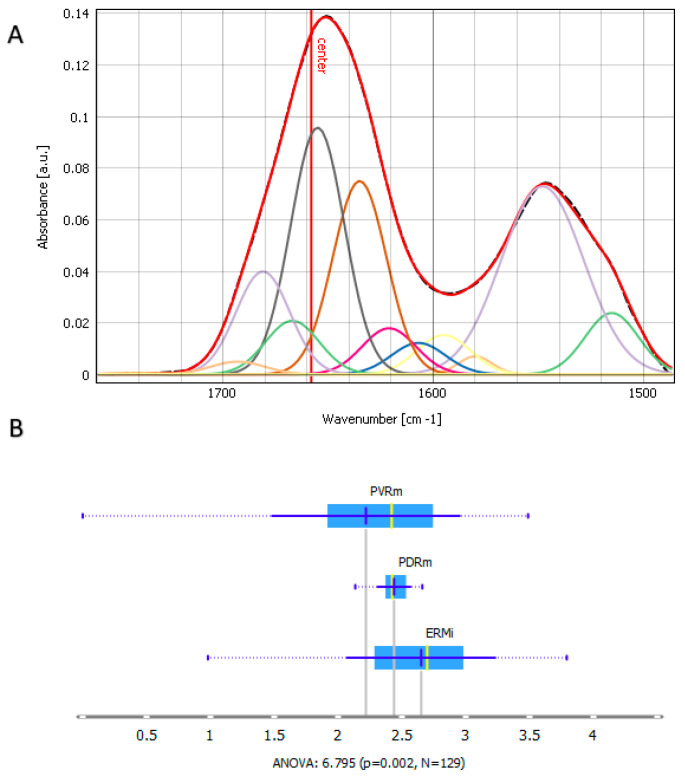
Band deconvolution of the Amide I and II and carbonyl region (1480–1760 cm^−1^). (**A**) Deconvoluted spectra with Gaussian curves. The number of curves and Gaussian curves’ maxima positions have been obtained from the article by Kreuzer et al., 2020. In the fitting procedure, the curve wideness of Amide I has been fixed to 30 cm^−1^. (**B**) Box plots of the areas below each Gaussian curve for ERMi, PVRm and PDRm, representing *α*-helix (1658 cm^−1^). Values are presented with the probability density and mean +/− SD. Analysis of variance (ANOVA) is used to test the difference between three means, where the *p*-value represents the probability of the occurrence of a given event and the N is the number of the measured individual areas/spectra that were analyzed.

With the aim of analyzing the discovered changes in the secondary protein structure in ERMi, PVRm, and PDRm more thoroughly, the Amide I and II bands have been deconvoluted using 12 Gaussian functions. The Gaussian curves’ maxima positions have been approximated in accordance with the article by Kreuzer et al., 2020 [19]. Figure 3A reveals the deconvolution of a single spectrum as an example. The allocation of the identified bands within the Amide I band (1600–1700 cm^−1^) was performed utilizing formerly described spectral components connected with different secondary protein structures [19,20,21]. The bands correlating with the region 1605–1620 cm^−1^ are assigned to side chains; 1620–1630 cm^−1^ to cross β-sheets; 1630–1637 cm^−1^ to parallel β-sheets; 1638–1646 cm^−1^ to unordered structures; 1647–1662 cm^−1^ to α-helices; 1662–1678 cm^−1^ to loops and turns; and 1690–1697 cm^−1^ to anti-parallel β-sheets. Additionally, the band assigned to the carbonyl group between 1730–1760 cm^−1^ has also been analyzed. The bands of the Amide II group at 1548 cm^−1^ and 1515 cm^−1^ are attributed to α-helices (Figure S1) and Tyrosine (Figure S1), respectively [22]. This analysis was focused on the Amide I band. The areas under all Gaussian peaks have been integrated for each spectrum and shown as box plots for ERMi, PDRm, and PVRm (Figure S1). Additionally, the box plot for α-helix is shown in Figure 3B. Interestingly, α-helix showed the highest expression in ERMi and the lowest expression in PVRm.

Regarding Amide I, the deconvolution showed that the difference between ERMi, PDRm, and PVRm is the most noticeable for the turns and loops secondary structure with the peak maximum at 1666 cm^−1^ being the most expressed in PDRm and the less expressed in PVRm (Figure S1). PVRm also had the lowest expression of α-helices in Amide II (Figure S1), β-sheet (Figure S1) and oligomers (Figure S1). The analysis further confirmed that in PDRm, there is the lowest presence of Tyr (Figure S1) and side chain as Tyr, Glu, and Asp residues (Figure S1), which showed the biggest expression in PVRm. In PDRm, there is also the lowest presence of side chains such as Tyr and Asn (Figure S1), turns and loops with the peak maximum at 1681 cm^−1^ (Figure S1), and a carbonyl group (Figure S1), which showed the biggest expression in ERMi. In ERMi, there is also the biggest expression of α-helices in Amide II (Figure S1).

### 2.4. Nucleic Acids and Carbohydrates Regions

**Figure 4:**
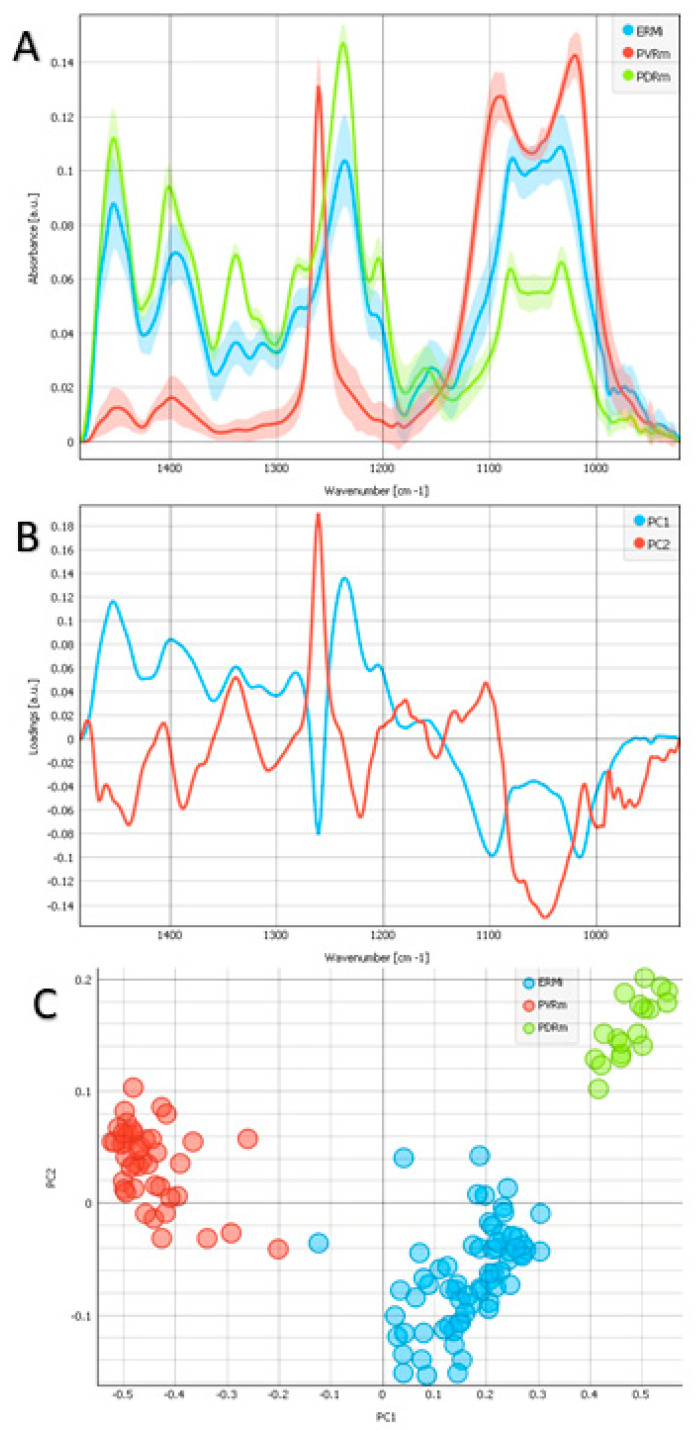
Analysis of the spectral region corresponding to the nucleic acids and carbohydrates in the wavenumber region (950–1485 cm^−1^). Average spectra (**A**) of examples of 3 different types of epiretinal proliferations (ERMi-blue, PVRm-red, PDRm-green) and corresponding loadings plot (**B**) with PC1 (blue) and PC2 (red). The PCA scores (**C**) plot denotes the variability associated with the first two components.

The wavenumber region between 950 and 1485 cm^−1^ corresponds to the nucleic acids and carbohydrates (Figure 4A) [17,18]. Besides the common bands of biological samples, the spectra of PVRm show a very pronounced peak with a maximum of 1261 cm^−1^. This peak is connected to the CH_3_ symmetric deformation of polydimethylsiloxane (PDMS) [23]. In addition, PDMS demonstrates two strong bands at 1090 cm^−1^ and 1022 cm^−1^, corresponding to Si–O–Si stretching vibrations. The peak at 1033 cm^−1^, associated with the sugar rings in carbohydrate residues in collagen and proteoglycans [24], shows a stronger expression in ERMi and a weaker expression in PDRm.

The scores plot demonstrates that the groups strongly separate along PC1 (Figure 4C). The PC1 loadings have contributions at 1237 cm^−1^ (maximum), 1454 cm^−1^ (maximum), 1261 cm^−1^ (minimum), and 1098 cm^−1^ (minimum) (Figure 4B). The first maximum corresponds to PO_2_- asymmetric stretch in deoxyribonucleic acid (DNA) and the second to the CH_2_, CH_3_ deformation modes in proteins. They are more expressed in PDRm than in ERMi, with very low expression in PVRm. The minima correspond to PDMS and are strongly expressed in PVRm.

### 2.5. Additional Protein Analysis

**Figure 5:**
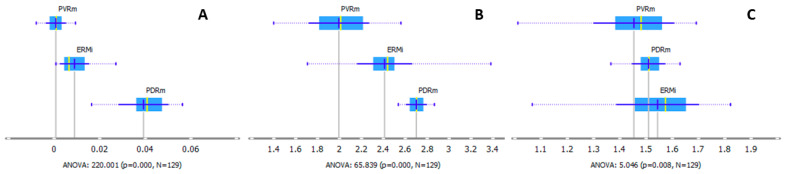
Analysis of the (**A**) collagen concentration (1338 cm^−1^), (**B**) spectra for the collagen maturity (1660 cm^−1^/1690 cm^−1^), and (**C**) spectral changes in the protein conformations by using the ratio of the protein C=O stretching of the structural protein (Amide I) to the N-H bending and C-N stretching of the polypeptides and protein background (Amide II) (1654 cm^−1^/1554 cm^−1^). Values are shown with the data probability density and mean +/− SD. Analysis of variance (ANOVA) is used to test the difference between three means, where the *p*-value represents the probability of the occurrence of a given event and the N is the number of the measured individual areas/spectra that were analyzed.

The peak at 1338 cm^−1^ corresponds to the amount of collagen present [25,26,27]. The intensities have been compared after a baseline correction of the single peak has been performed, according to Liu et al., 2006 [26]. The peak is the highest for PDRm (Figure 5A). We also analyzed the ratio of the areas for 1660/1690 cm^−1^ that corresponds to the degree of collagen maturity [24]. The ratio is the highest for PDRm and the lowest for PVRm (Figure 5B). The band ratio inspecting the spectral changes in the protein regions 1654 cm^−1^/1554 cm^−1^ has also been analyzed. The absorbance peak at 1654 cm^−1^ corresponds to the protein C=O stretching of the structural protein (Amide I), while 1554 cm^−1^ corresponds to the N-H bending and C-N stretching of the polypeptides and protein background (Amide II) [28]. ERMi has the highest ratio, and PVRm has the lowest (Figure 5C).

## 3. Discussion

Chemical alterations precede and/or accompany morphological changes that are symptomatic of disease [29]. FTIR is a method that enables the analysis of the biochemical status and differences in the molecular composition of tissue, cells, and ECM. We were interested in finding the possible differences between three different types of pathological tissue on the surface of the retina: ERMi, PVRm, and PDRm. Here, we did a pilot study in evaluating bio-macromolecules and detecting and assigning the proteins’ conformational changes, and also changes in collagens, lipids, carbohydrates, and nucleic acids in the three samples. It was recently indicated that FTIR is a helpful analytical technique for the analysis of ERMs and that it allows the analysis of the spatial distribution of protein secondary structures in the ERMs [13]. Unlike the mentioned study, we utilized SR-FTIR with high spatial resolution. SR-FTIR micro-spectroscopy is also a powerful method for a fast and thorough chemical structural characterization of collagen characteristics from various origins, including different natural and synthetic collagens [30].

Biochemical differences connected with lipid metabolism can be recognized by the investigation of the high-wavenumber region, with the bands that are associated with the asymmetric (ν_as_) and symmetric (ν_s_) methyl (CH_3_) and methylene (CH_2_) groups’ stretching vibrations detected at ~2960 [ν_as_(CH_3_)], 2923 [ν_as_(CH_2_)], 2873 [ν_s_(CH_3_)], and 2850 cm^−1^ [ν_s_(CH_2_)] [17]. We discovered that the asymmetric and symmetric CH_2_ vibrations are more expressed in ERMi. PDRm showed the strongest contribution in the asymmetric CH_3_ vibration. The CH_2_ bands are mostly because of the saturated chains in lipids, whereas the CH_3_ bands are because of the vibrations of the methyl groups in proteins, lipids, and nucleic acids [17]. A high lipid concentration arrives from plasma membranes and microsomal pellets such as endosomes and secretory vesicles, whilst just a low lipid concentration arrives from the cytoplasm [17]. The vibration of CH_2_ was the most expressed in ERMi, suggesting a higher lipid concentration, and it shifted toward higher wavelengths for PDRm. The CH_2_ groups’ symmetric stretching mode location can be a pointer to the lipid-membrane’s order and disorder that is influenced by the content, composition, and hydration of membrane proteins [31]. In such instances, a helpful parameter can be the shift in the CH_2_ stretching vibration’s wavenumber, being the case for PDRm that point to alterations in acyl chain flexibility [32]. The shifting towards lower frequencies can also point to an augmentation in the number of trans isomers of lipids or a different lipid milieu that can be associated with an augmentation in lipid order and chain rigidity [33].

The lipid region of the PVRm spectra was pronouncedly different from the other epiretinal proliferations with the biggest expression at 2962 cm^−1^ [ν_as_(CH_3_)], asymmetric CH_3_ vibration. The appearance of the PVRm spectrum was surprisingly very much like a combination of polydimethylsiloxane (PDMS) [23] with common biological tissue. PDMS, also known as dimethylpolysiloxane, is one of several types of polymerized siloxanes or silicone oil (SO), which has wide applications in industry, medicine, and cosmetics. SOs for ophthalmic use, also named organosiloxane, are synthetic polymers composed of PDMS with different chain lengths [34]. They are constituted of silicon–oxygen bonds and have hydrocarbon radicals as radical side groups [35]. The purpose of the use of SO in vitreoretinal surgery is to serve as either a short-term or long-term endo-tamponade of the retina in complicated vitreoretinal diseases, such are complex retinal detachments and PDR [34,35]. In our case with retinal detachment and PVR, SO was also used as an intraocular tamponade implant. Despite the advantages and the fact that SO has been in use in ophthalmology for half a century, there have been constant concerns about the complications that arise when using it. Most of the complications are related to the tendency of SOs to emulsify [35]. The emulsified droplets have the potential to move to different intraocular structures. These emulsified particles have been shown to stimulate macrophages and other proliferating cells within proliferative ERMs, leading to their impregnation and vacuolization [36,37]. The process of formation and the confluence of intracytoplasmic vacuoles is believed to occur by the mechanism of endocytosis of SO droplets, mainly by macrophages and cells of glial origin [37]. A very dissimilar appearance of proliferating cells in PVR membranes on transmission electron microscopy with membrane-bound vacuoles has been described by different authors earlier [36,37,38,39,40]. Although the presence of emulsified SO in intracytoplasmic cell vacuoles in PVR membranes has not been directly proven, it was predicted on the basis of indirect facts [37]. We believe that the results of our analysis, for the first time, demonstrate the presence of SO or PDMS in the structure of a PVR membrane, which confirms previous assumptions that cells in PVRm formed in patients with RRD and tamponade with SO are vacuolated and that vacuoles are filled with SO. However, the results have to be verified by increasing the study group and probing a larger number of PVRm samples.

The impact of PDMS must be taken into consideration when interpreting the different wavenumber regions of the PVRm spectra. Although PVRm spectra show the contribution of the PDMS, PVRm spectra differ from the PDMS spectra in the region from 1300 cm^−1^ to 1700 cm^−1^ [23], which hence belongs to the membrane itself. Therefore, in the Amide I and Amide II regions, there is the contribution mostly from the biological tissue as part of the PVRm. As a result of the high impact of PDMS in the spectra of PVRm, it was possible to compare three types of membranes only for the Amide region but not for the lipids, oxidative stress, and carbohydrate regions.

Our results show that the Amide I band maxima were found at 1650 cm^−1^, 1653 cm^−1^, and 1657 cm^−1^, and Amide II bands maxima at 1543 cm^−1^, 1547 cm^−1^, and 1553 cm^−1^ for PVRm, ERMi, and PDRm, respectively, indicating differences in the secondary structure content of all three membrane types (Figure 2A). The band deconvolution showed the presence and the individual contributions of the α-helix (1658 cm^−1^*)* for the different membranes (Figure 3B).

A characteristic of the connective tissue is the absorbance triad in the region of the Amide III vibrations, i.e., 1206 cm^−1^, 1238 cm^−1^, and 1280 cm^−1^ coming mostly from collagen [17]. Our results, based on the bands at 1204 cm^−1^ (Figure 4A) and 1338 cm^−1^ (Figure 5A), suggest the strongest collagen deposition in PDRm, less in ERMi, and absent in PVRm.

Ratiometric analysis on the spectra for each type of membrane was performed for the collagen maturity (1660 cm^−1^/1690 cm^−1^) [24] (Figure 5B) and protein conformation (1654 cm^−1^/1554 cm^−1^) [28] (Figure 5C). Differences in the protein conformations—calculated by utilizing the ratio of the protein C= O stretching of the structural protein (Amide I) to the N-H bending and C-N stretching of the polypeptides and protein background (Amide II)—may correlate with the alterations in the structural reorganization of present proteins or the new proteins’ expression with various structural properties. These alterations are believed to be because of the deposition of ECM proteins like collagen, fibronectin, and laminin in the course of fibrosis development [28]. The upregulated ECM collagen-like proteins in ERM and PDR were demonstrated in the study by George et al. [1]. Using the ratio 1654 cm^−1^/1554 cm^−1^, we found the strongest changes in protein conformations in ERMi, smaller in PDRm and the smallest in PVRm (Figure 5C). We showed that the PDRm contained more β sheets than ERMi, suggesting the higher content of type IV collagen in PDRm (Figure S1). Elevated levels of collagen type IV in PDR membranes have been previously demonstrated by immunohistochemical analysis together with its upregulation by molecular methods [1,9]. The existence of collagen types I, type IV, entactin, and fibronectin in ERMi has been demonstrated by Altera et al., with immunofluorescence and confocal microscopy [41].

The band at 1033 cm^−1^ is allocated to the sugar rings in carbohydrate residues in collagen and proteoglycans [24]. We found its stronger presence in ERMi and weaker presence in PDRm (Figure 4A). It has been shown that growth in the intensity of the bands at 1080 cm^−1^ and between 990 cm^−1^ and 970 cm^−1^ mirrors continually raised the phosphorylation of proteins [17]. We found it at a higher level also in ERMi and less in PDRm (Figure 4A). Therefore, glycoprotein- and collagen-associated carbohydrate moieties were present in ERMi and less in PDRm (Figure 4).

Even though the collagens’ and cells’ FTIR spectra are thought to be highly analogous, the existence of different types and configurations of proteins in cells, as well as other types of macromolecules, may alter the positions and shapes of the Amide bands [42]. The band at 1454 cm^−1^ is assigned to the CH_2_ and CH_3_ deformation modes and asymmetric methyl deformation, represented in proteins [43]. We found the higher expression in PDRm, lower in ERMi, and the lowest for PVRm (Figure 5A). The PO2- asymmetric stretch at 1237 cm^−1^, which represents DNA [44], showed the highest expression in PDRm, lower in ERMi, and it was absent in PVRm (Figure 4A), suggesting possible higher cell content in PDRm.

This study has some limitations. The main limitation is the small number of samples. However, although we have analyzed a small number of samples, the differences between them are very pronounced and specific to each type of membrane, which is a novelty and a good basis for further investigations. Another limitation is that three different membranes taken from the eye were all stored in PFA. Nevertheless, the differences among them were clear and could be compared regardless of the PFA treatment. The tissue preparation methods’ importance for biological tissues’ FTIR micro-spectroscopical analysis is discussed in Zohdi et al., 2015 [45]. Two characteristic bands of collagen at 1205 cm^−1^ and 1285 cm^−1^ were noticeable in the wet hearts’ tissue spectra but were only weakly revealed in the formalin-fixed and ethanol-dehydrated tissue spectra and were lacking from the desiccated sample spectra [45]. In our spectra, the shoulders for 1205 cm^−1^ and 1285 cm^−1^ were observed for PDRm and with smaller intensity for ERMi but not for PVRm (Figure 4A).

## 4. Materials & Methods

### 4.1. Sample Preparation

The membranes were collected from routine pars plana vitrectomy performed at the Eye Hospital, University Medical Centre, Ljubljana, Slovenia. The sample collection complied with the tenets of the Helsinki Declaration. Informed consent was provided before surgery for each patient.

In the case of a 75-year-old female with ERMi, the surgery was indicated due to deterioration of visual acuity and metamorphopsia. The membrane was obtained during uneventful surgery. The epiretinal tissue was peeled off by using microforceps. PDRm was obtained from a 65-year-old male with PDR undergoing vitrectomy due to vitreous hemorrhage and tractional retinal detachment. PVRm was obtained from a 63-year-old male after surgery with 2000 centistoke silicone oil (SO) endotamponade due to RRD and PVR stage C6. SO was removed 6 months after surgery. During the same procedure, PVRm was peeled off using microforceps. The removed tissue was, in all cases, immediately stored in paraformaldehyde (PFA).

Each membrane was washed after collection in a high-glucose medium (DMEM; Sigma, no. 5671, St. Louis, MO, USA) supplemented with 10% fetal bovine serum (FBS) and 1% antibiotics (penicillin–streptomycin; Sigma, no. 4333). ERMs were then prepared for FTIR micro-spectroscopy studies: firstly, being rinsed in 5 mL NaCl for 10 min and then, by using microdissecting tweezers (WPI by Dumont, Med. Biologie, Germany), were placed by gently stretching and plating adherently on circular 13 mm *×* 0.5 mm calcium fluoride, CaF_2_ slides (Crystan Ltd., Dorset, UK). After this, the samples were dried under sterile conditions in the laminar flow at room temperature and then stored with silica gel until the measurements at the ALBA synchrotron.

### 4.2. Synchrotron Radiation-Based FTIR Micro-Spectroscopy

For the purpose of assessing the organic compounds’ profiles, we carried out measurements at the infrared micro-spectroscopy beamline MIRAS at the ALBA synchrotron light source (Barcelona, Spain) [46] using SR-FTIR micro-spectroscopy. Conventional FTIR spectroscopy is a helpful tool for investigating larger cell populations in the tissues. However, the restricted standard infrared light sources’ brightness generally prevents measurements with high spatial (single-cell) resolution in comparison with SR-FTIR micro-spectroscopy [18]. All SR-FTIR micro-spectroscopic absorption spectra were collected in transmission mode using the infrared microscope Hyperion 3000 coupled to a Vertex 70 spectrometer (Bruker, Germany), equipped with a liquid nitrogen-cooled mercury cadmium telluride detector and the mid-infrared region of the synchrotron light as the infrared light source. Each spectrum was acquired after co-adding 128 scans at a spectral resolution of 4 cm^−1^. OPUS Version 8.2 (Bruker, Germany) software package was used for data collection.

**Figure 6:**
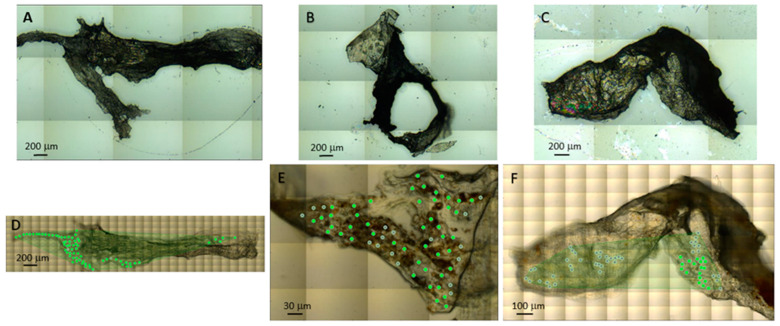
Visible image of ERMi (**A**,**D**), PVRm (**B**,**E**) and PDRm (**C**,**F**) postoperative tissue obtained in reflection geometry (**A**–**C**) and transmission geometry (**D**–**F**) with higher magnification, achieved with Schwarzschild objectives; green dots represent the measured areas of the spectra used in the analysis, grey dots represent the measured areas of the spectra not used in analysis.

For the purpose of achieving the single-cell data acquisition and analysis, we obtained the spectra of 10 *×* 10 µm^2^ areas of the tissue by using the microscope’s aperture and the highly focused synchrotron source’s infrared light. Visible images of the postoperative tissue of ERMi, PVRm, and PDRm were obtained in reflection geometry (Figure 6A–C) and transmission geometry (Figure 6D–F), the latter also showing the measured points. Spectra with infrared absorbance higher than 2, at the wavenumbers 1650 cm^−1^ and 1020 cm^−1^, were not considered in the analysis. In total, 129 measured individual areas/spectra were analyzed: 67, 44, and 18 individual areas/spectra from the measured (71, 63 and 71) individual areas/spectra from the ERMi, PVRm and PDRm, respectively.

The spectral analysis was concentrated on three regions of the spectra: (950–1485 cm^−1^), i.e., nucleic acids and carbohydrates, and Amide I and II (1485–1760 cm^−1^), i.e., proteins and lipids (2800–3100 cm^−1^). In the regions of interest, spectra were baseline-corrected, and unit vectors were normalized. Quasar 1.3.0 software package (Bioinformatics Laboratory of the University of Ljubljana, Version 3.20.1) with the spectroscopy package [47] was used for data correction and further analysis. Principal component analysis (PCA) was used for the data sets’ comparison and concentrated on the first two principal components. In addition, Amide I and II bands and carbonyl region (1480–1800 cm^−1^) were deconvoluted for each spectrum by using Quasar 1.3.0. with the spectroscopy add-on Peak Fit. The region was fitted using 12 Gaussian functions for each spectrum. The center Gaussians’ positions have been approximated from the article by Kreuzer et al., 2020, while 1580 cm^−1^ was included to get a better baseline [19]. The center positions were fixed. For all Gaussians regarding Amide I, the full wideness at half maximum was set fixed to 30 cm^−1^ (σ = 12.77 cm^−1^). The resulting areas underlying the Gaussian bands were calculated for each spectrum and plotted in boxplots. Values are shown with the data’s probability density and mean +/− SD.

## 5. Conclusions

We have already used SR-FTIR to study the lens epithelial cells’ macromolecular compounds in human cortical and nuclear types of cataracts [19] as well as the UV effect on human anterior lens capsule macro-molecular composition [48]. The present results give further evidence that SR-FTIR is sensitive to the pathologic processes of epiretinal proliferations. Each of the studied samples showed a different spectral profile, which suggests a different macro-molecular composition of three different types of epiretinal proliferation originating from different constituents and metabolic processes is taking place. PVRm differs the most from ERMi and PDRm in all spectral regions. Further studies on a larger number of samples and also samples in different stages of evolution are needed. With this work, we hope to stimulate further investigations.

Different ultrastructural studies have shown the complex molecular structure of epiretinal proliferations. The presence of a variety of cells and ECM composed of proteoglycans and fibrous proteins such as collagen, fibronectin, elastin, and laminin has been demonstrated in previous reports. We believe that part of the described SR-FTIR signal in our samples is coming from collagen, as collagen constitutes up to 30% of total protein mass, being the most abundant protein in ECM. In addition to the PDMS presence in PVRm—which is the most characteristic finding in our sample of this type of retinal proliferation—we were able to identify differences between samples of PDRm and ERMi in lipid structure, collagen content and collagen maturity, differences in proteoglycan presence, protein phosphorylation, and DNA expression. Our results suggest that collagen is most present in PDRm.

## Data Availability

The data presented in this study are available at request from the corresponding author.

## Supplementary Materials

The supporting information can be downloaded at: https://www.mdpi.com/article/10.3390/ijms24054834/s1.
