# Supplementary material for: Characterization of Different Types of Epiretinal Proliferations by Synchrotron Radiation-Based Fourier Transform Infrared Micro-Spectroscopy
Source: Int J Mol Sci. 2023 Mar 2;24(5):4834. doi: 10.3390/ijms24054834 (PMC10003457; doi:10.3390/ijms24054834)
Supplement: Supplementary file 1 [file ijms-24-04834-s001.zip › ijms-2196889-supplementary.pdf]

Supplementary file

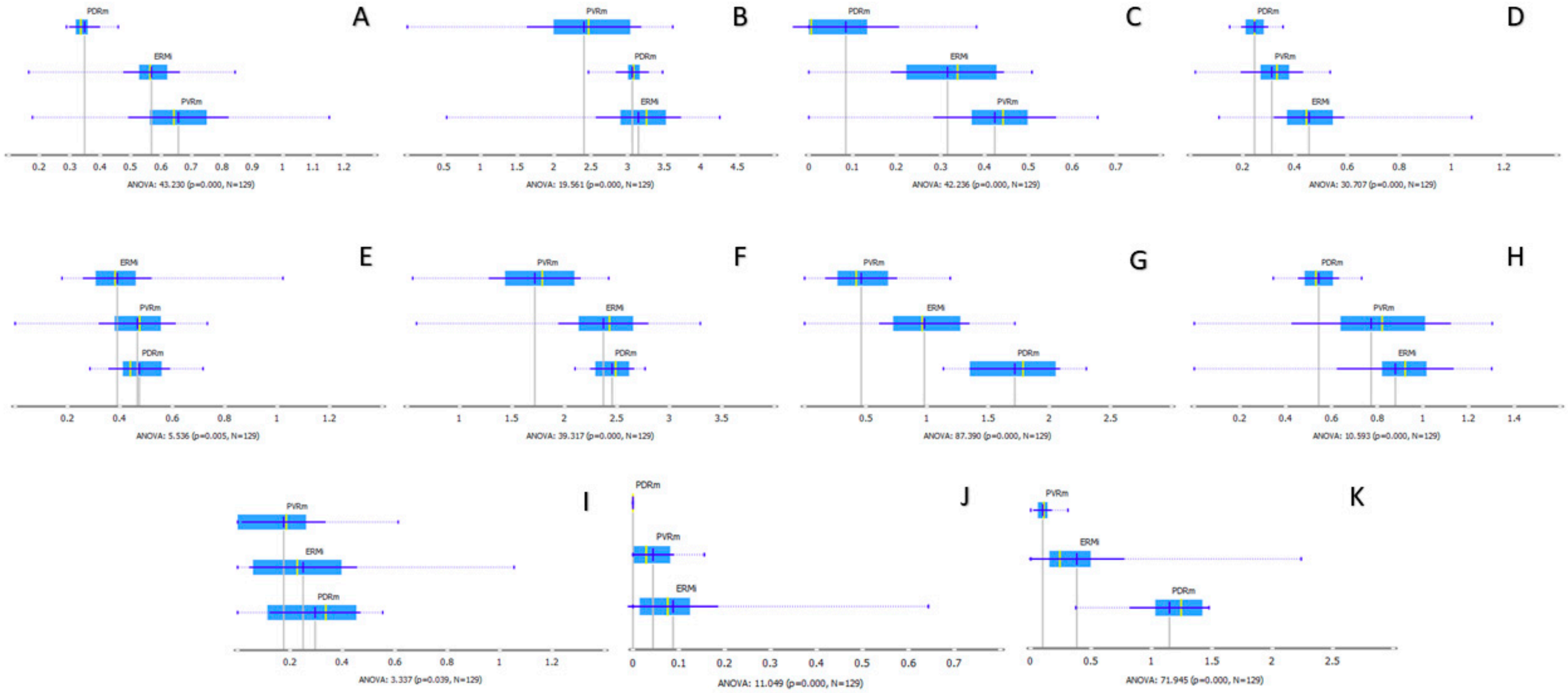

Figure S1. Box plots of the areas below each Gaussian curve for ERMi, PVRm and PDRm, representing: a) Tyr (1515 cm^{-1}), b) α-helices in Amide II (1548 cm^{-1}), c) side chain as Tyr, Glu, and Asp residues (1595 cm^{-1}), d) side chains such as Tyr and Asn (1610 cm^{-1}), e) cross-β-sheet (1625 cm^{-1}), f) β-sheet (1635 cm^{-1}), g) and h) turns and loops (1666 cm^{-1} and 1681 cm^{-1}), i) oligomers (1693 cm^{-1}), and j) carbonyl group (1740 cm^{-1}). k) 1580 cm^{-1} was added to achive a better baseline. Values are presented with the probability density of the data at different values and mean +/-SD.
